# The use of Shewhart control charts when conducting cavitation studies to identify and eliminate non-random causes of variability

**DOI:** 10.1016/j.mex.2024.102929

**Published:** 2024-09-04

**Authors:** Mikhail Anatolyevich Samarin, Nikita Andreevich Shostak, Etibar Yusif Oglu Balayev, Anahit Seiranovna Basyuk

**Affiliations:** Institute of Oil, Gas and Energy, Kuban State University of Technology, Krasnodar, 350072, Russia

**Keywords:** The method of using Shewhart control charts during cavitation studies to identify and eliminate non-random causes of variability, Cavitation erosion, Cavitation, Shewhart control charts, Causes of process variability, Statistical methods, Ultrasonic transformations

## Abstract

One of the processes that accompanies the operation of various hydraulic systems is cavitation. This phenomenon is often accompanied by cavitation erosion, that is, the progressive loss of parent material from a solid surface due to continued exposure. The problem of obtaining accurate and reliable data when conducting cavitation studies remains relevant. This article discusses the adaptation of the use of Shewhart control charts when conducting cavitation studies, in order to determine the presence of non-random “special” causes of variability. A graph of changes in the process parameters over time was constructed to carry out statistical control of the stability of the process in order to determine the boundaries of the system variability of the process in order to predict the behavior of the process. As a result of the conducted research, recommendations were developed to increase the accuracy of output data when conducting cavitation studies. It has been confirmed that the use of control charts as a tool for quality control of laboratory measurements allows us to establish that the process has achieved a statistically controlled state, which allows us to maintain a high degree of stability and quality of the research being carried out.

This method makes it possible:

• to determine the presence of non-random “special” causes of variability during cavitation studies.
• to quickly identify and eliminate the “special” causes of variability during cavitation studies.

## Graphical abstract

**Figure:**
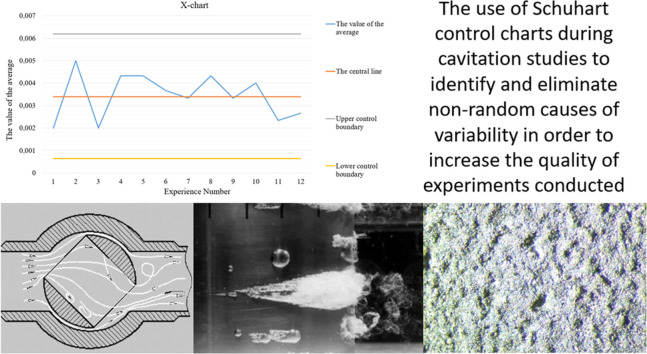

Specifications table

**Table:** 

Subject area:	Mathematics and Statistics
More specific subject area:	Statistical control
Name of your method:	The method of using Shewhart control charts during cavitation studies to identify and eliminate non-random causes of variability
Name and reference of original method:	Shewhart WA. Economic control of quality of manufactured product. New York: D. Van Nostrand. 501 p. 1923. ASTM G32–16(2021)e1 Standard Test Method for Cavitation Erosion Using Vibratory Apparatus
Resource availability:	A laboratory ultrasonic complex model LUK-0.5/20-O LLC Center for Ultrasonic Technologies; Microsoft Office Excel

## Background

Cavitation accompanies the operation of various hydraulic systems. The essence of the phenomenon is the formation and subsequent compression of cavities (bubbles) inside the liquid containing steam or a mixture of steam and gas. This phenomenon is often accompanied by cavitation erosion, that is, the progressive loss of parent material from a solid surface due to continued exposure. Despite the fact that this phenomenon was discovered quite a long time ago, its research is still ongoing [[1], [2], [3], [4], [5], [6], [7], [8]]. Today, the only standard regulating the conduct of cavitation studies, that is, studies of the resistance of solid materials to cavitation erosion, is c. This standard was introduced in 1998 (ASTM G32–98) and has been regularly updated and updated since then. The method given in this standard [4] is widespread and used when carrying out research work related to determining the resistance of materials and coatings to cavitation by creating cavitation bubbles with ultrasonic vibrations. At the same time, despite the fact that this method has been sufficiently studied, the issue of increasing the reliability and accuracy of the data obtained during testing remains relevant. Taking into account the lack of complete understanding of the laws of the process of cavitation erosion, as well as taking into account the existing experience, we can conclude that cavitation is a phenomenon dependent on many factors, which requires a special approach when conducting cavitation studies.

Almost all research conducted in laboratories is processed using statistical methods in order to increase reliability. There are a significant number of these methods. Some of them are aimed at obtaining mathematical dependencies of output parameters on input ones, and some are aimed at assessing the received data.

One of the ways to assess the reliability and convergence of output data is the use of Shewhart control charts. This method is a graphical way to assess the degree of statistically uncontrollable state of a process by comparing individual statistical data from a series of samples or subgroups with control limits [[9], [10]].

This method was first introduced in 1924 by Walter Shewhart with the goal of reducing process variability by eliminating deviations caused by non-systemic causes [[10], [11], [12], [13], [14], [15], [16], [17], [18], [19], [20], [21], [22], [23], [24], [25], [26], [27]]. This method has proven itself and is used in most enterprises to manage production and business processes, to identify instability, allowing to obtain a controlled process. In addition to use in industrial enterprises, this method is applicable when carrying out research work. The use of this method makes it possible to assess the degree of stability of the tests being carried out and to identify patterns in unstable data. At the same time, it is worth noting that this method, due to its versatility, has no restrictions if it is correctly applied in accordance with existing standards.

The purpose of this article is to adapt the use of Shewhart control charts when conducting cavitation studies, as well as to develop recommendations for increasing the accuracy of output data when conducting cavitation studies in order to ensure the possibility of effectively detecting the influence of special causes on the process (tool breakage; improper adjustment; equipment wear; insufficient uniformity of the processed material; errors in control and measuring equipment; fluctuations in energy sources; environmental changes, etc.) and its exit from a statistically controlled state, which, in Ultimately, it will help improve the quality of the research being conducted. At the same time, it is worth noting that this article does not provide a new modification of Schuhart's control charts, but describes a new way of using it in experimental conditions.

## Method details

### Experimental and computational part

An interdisciplinary team of researchers from the Laboratory of Advanced Design of Oil and Gas Equipment and Technologies of the Federal State Budgetary Educational Institution of Higher Education “Kuban State Technological University” conducted studies of the cavitation resistance of some steels used in various industries. The essence of the above research method is to create cavitation damage on the surface of the sample by creating high frequency vibration when immersed in liquid. This method uses an ultrasonic transducer in which a special element made of the material being studied is installed. The studies were carried out according to the ASTM G32–16(2021)e1 standard. A laboratory ultrasonic complex model LUK-0.5/20-O LLC Center for Ultrasonic Technologies was used as a transducer. The operation of the device is based on the principle of electronic conversion of electrical network energy into mechanical ultrasonic vibrations using the piezoelectric effect. The frequency of mechanical vibrations was 20±0.5 kHz, power – 100 %, ambient temperature 20± 5°С, which corresponds to the above standard.

Before each new test, the liquid was replaced, which was water. The test sample was weighed on an analytical balance with an accuracy of 0.001 g. Special “button patches” were used as samples, installed on the horn of the ultrasonic installation using the existing threaded element. The sample must fit snugly against the horn, which is achieved by tightening it. The device is set to 100 % power and a 1 hour timer. One sample was tested 12 times, that is, total cavitation erosion occurred over 12 h. The sample is immersed in water to a depth of 8 to 16 mm. After the test, the sample is unscrewed from the horn of the ultrasonic unit, cleaned and weighed on a scale. Then, the surface of the sample subjected to cavitation erosion is additionally examined using a microscope in order to identify patterns of cavitation wear of the surface.

AISI 304 steel was used as samples [28]. The results of measurements of mass wear of samples are presented in Table 1. The initial masses of samples No 1, No 2 and No 3, respectively (in grams): 8742; 8746; 8745.

**Table 1:** Results of measurements of mass wear of samples and associated statistical data.

Experiment number	Change in mass of sample No 1, g	Change in mass of sample No 2, g	Change in mass of sample No 3, g	Subgroup range R, g	Subgroup mean $\bar{X}$, g	Sample standard deviation s, g
1	0,001	0,002	0,003	0,002	0,0020	0,0010
2	0,005	0,006	0,004	0,002	0,0050	0,0010
3	0,002	0,003	0,001	0,002	0,0020	0,0010
4	0,004	0,004	0,005	0,001	0,0043	0,0006
5	0,006	0,001	0,006	0,005	0,0043	0,0029
6	0,004	0,005	0,002	0,003	0,0037	0,0015
7	0,005	0,002	0,003	0,003	0,0033	0,0015
8	0,003	0,006	0,004	0,003	0,0043	0,0015
9	0,003	0,006	0,001	0,003	0,0033	0,0025
10	0,002	0,004	0,006	0,004	0,0040	0,0020
11	0,002	0,001	0,004	0,003	0,0023	0,0015
12	0,003	0,002	0,003	0,001	0,0027	0,0006

The table also shows the range of the subgroup (the difference between the largest and smallest observations in the subgroup), the mean of the subgroup and the sample standard deviation calculated using formula 1.

(1) $$s=\;\sqrt{\frac{\sum {\left(X_{i}-\bar{X}\right)}^{2}}{n-1}}$$

Experimental and computational part

Before constructing Shewhart control charts (this article considers charts of averages X, ranges R, or sample standard deviations s), it is necessary to determine control limits (Table 2) and coefficients for the parameters of the corresponding control charts. Formulas and coefficients for their determination were taken from ISO 7870–2:2013.

**Table 2:** Assessment of control limits.

Statistics	Estimates of control limits	
	Central line, g	Control boundaries, g
$\bar{X}$	$\bar{\bar{X}}$ = 0,0034	$\bar{\bar{X}}\;\pm A_{2}\bar{R}$*=* 0,0062/0,00,064
R	$\bar{R}$ = 0,0027	$D_{4}\bar{R}$ = 0,0069
s	$\bar{s}$ = 0,0015	$B_{4}\bar{s}$ = 0,0038

X and R range charts are used when the sample size is small or moderate (usually <10). In the case of large subgroup sample sizes (*n* > 10), the use of X and s charts are preferable, since as the sample size increases, the range becomes less effective as an estimate of the standard deviation of the process. If electronic devices are used to calculate process boundaries, it is preferable to use standard deviation.

**Fig. 1:**
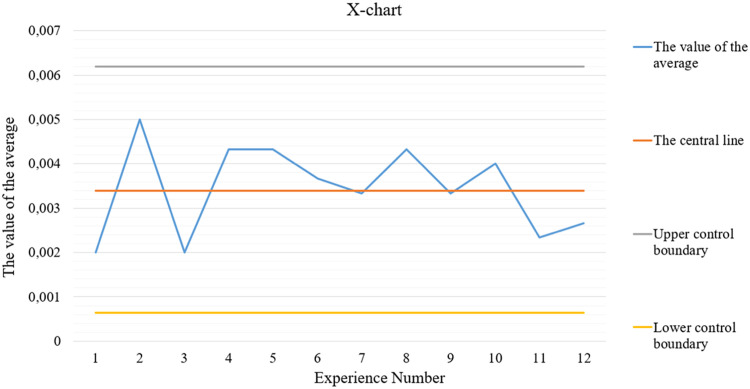
X-chart.

**Fig. 2:**
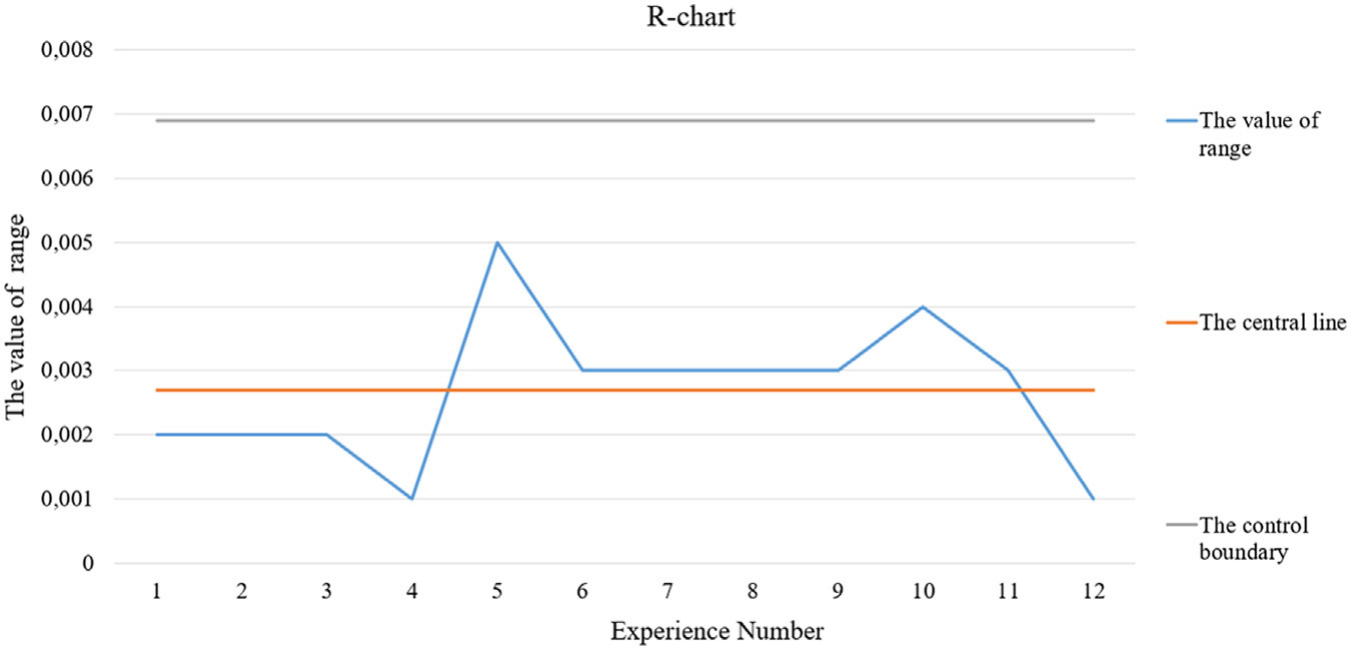
R-chart.

**Fig. 3:**
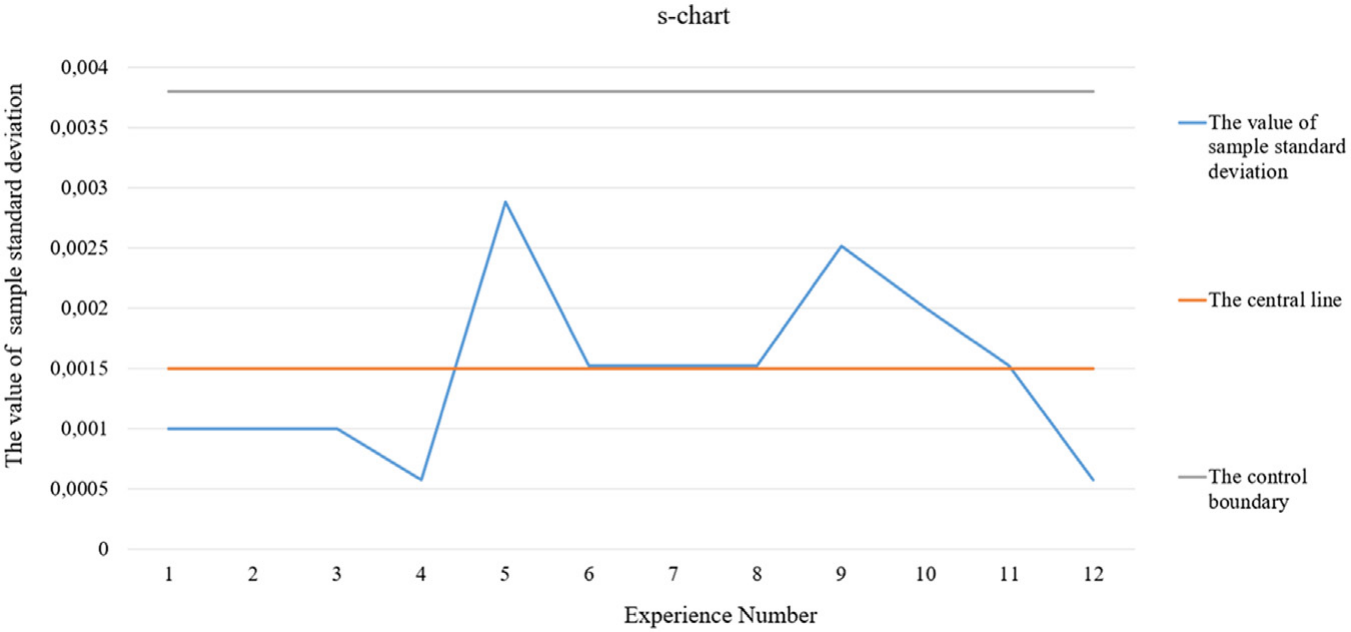
s-chart.

Shewhart's X, R, and s charts are shown in Figs. 1, 2, and 3, respectively.

## Conclusion

Based on the analysis of all three Shewhart control charts, we can conclude that the studies conducted are statistically controlled, which indicates that there is no need to adjust the experimental conditions and that the influence of “special” (or unnatural, systematic, controlled) causes of variability is quite low (variability): material heterogeneity, tool breakage, incorrect operation of production or control equipment, inappropriate personnel qualifications, violation of procedures, changes in laboratory conditions.

If Shewhart control charts show that the process is statistically uncontrollable (unstable), i.e. points on the graphs go beyond the control limits, then this should be perceived as a signal for the need to adjust the experimental conditions due to the influence of “special” reasons.

At the same time, Shewhart control charts can help detect non-random causes of process variability. For this purpose, GOST R ISO 7870–2–2015 in reference specifies criteria for identifying special causes. By carefully studying all the structures of points on the chart, which may indicate the impact of non-random causes on the process, it becomes possible to interpret typical structures using known criteria.

In order to increase the accuracy and reliability of the applied method for studying cavitation erosion, the team of authors developed the following recommendations that reduce the influence of controlled causes of variability

- it is necessary to replace the working fluid after each experiment, if possible, use an automatic fluid renewal system during the experiment - this will reduce the effect of temperature changes on the cavitation process (during the process of cavitation erosion, as well as the operation of the installation, a significant amount of heat will be released), and also reduce the influence of metal particles detached during cavitation erosion, the presence of which can intensify cavitation erosion with an increase in their concentration in the liquid;

- it is necessary to use distilled water, which will reduce the influence of the existing salts dissolved in the water;
- it is necessary to use special tools when screwing in and unscrewing the test samples in order to minimize their damage outside the experiment;
- it is necessary to control the immersion depth of the test sample, since the immersion depth changes the hydrostatic pressure acting on the surface of the sample, and, therefore, changes the conditions for the formation of cavitation bubbles;
- samples must be thoroughly cleaned after each test. It is recommended to use ethyl alcohol (the use of an ultrasonic cleaning bath is considered satisfactory), and the test sample must be thoroughly dried before weighing;
- installation of the test sample into the horn of the ultrasonic installation should occur in such a way that the inner surface of the horn and the outer surface of the sample are as dry as possible, and the tightening is sufficient to ensure the tightness of the connection, if liquid gets into the threaded connection during the cavitation study will begin to cavitate, which will lead to additional wear of the threaded and rear surfaces of the test sample, as well as premature wear of cavitation equipment;
- it is necessary to control the verticality of cavitation equipment; if there is a slight inclination of the test sample, cavitation erosion will occur unevenly, which can lead to instability of the data obtained;
- it is recommended to carry out cavitation studies without preliminary stops: in real conditions, the cavitation process usually occurs for a long time, which leads to hardening, which affects the cavitation resistance of the surface of the material; when cavitation studies are periodically stopped, relaxation of the material occurs, which leads to its softening, and it is important ensure that the equipment does not overheat;
- if there is a power scale indicator, it is necessary to control that fluctuations in the power level are not significant: if there are strong fluctuations in the power level of the device, testing must be stopped, since, most likely, this is due to equipment malfunctions or incorrect test mode.

Thus, the use of control charts as a tool for quality control of laboratory measurements allows us to establish that the process has achieved a statistically controlled state, which allows us to maintain a high degree of stability and quality of the research being carried out.

## Method validation

None.

## Limitations

None.

## CRediT authorship contribution statement

**Mikhail Anatolyevich Samarin:** Methodology, Software, Investigation, Data curation, Writing – original draft. **Nikita Andreevich Shostak:** Conceptualization, Validation, Writing – review & editing, Supervision, Project administration, Funding acquisition. **Etibar Yusif Oglu Balayev:** Methodology, Resources, Visualization. **Anahit Seiranovna Basyuk:** Validation, Formal analysis.

## Declaration of competing interest

The authors declare that they have no known competing financial interests or personal relationships that could have appeared to influence the work reported in this paper.

## Data Availability

Data will be made available on request.
